# Numerical simulation of cavitation threshold in water and viscoelastic medium based on bubble cluster dynamics^☆^

**DOI:** 10.1016/j.ultsonch.2025.107414

**Published:** 2025-06-02

**Authors:** Xiaozhuo Shen, Pengfei Wu, Weijun Lin

**Affiliations:** a State Key Laboratory of Acoustics, Institute of Acoustics and Marine Information, Chinese Academy of Sciences, Beijing 100190, China; b University of Chinese Academy of Sciences, Beijing 100049, China

**Keywords:** Cavitation threshold, Bubble cluster dynamics, Viscoelasticity

## Abstract

The ultrasonic cavitation threshold is a significant area of research in therapeutic ultrasound. This study conducts a numerical simulation of the ultrasound cavitation threshold by solving the dynamic equations of bubble clusters composed of bubbles with varying sizes. The effects of different criteria for cavitation threshold, bubble dynamics models, medium types, viscoelasticity and number of bubbles on the cavitation threshold are analyzed. Moreover, a comparison is made between calculation outcomes and those from previous experimental research. The results show that the relationship between the cavitation threshold *P* and frequency *f* can be expressed as *P* = A*f*^α^ *+* B, where A, B and α depend on the properties of the medium and different criteria of cavitation threshold. As the number of bubbles increases, the cavitation threshold initially rises and then falls, indicating a non-monotonic trend. Furthermore, the influence of the medium’s shear modulus on the cavitation threshold is more intricate, and the cavitation threshold in viscoelastic medium is not consistently higher than that in water. Among all parameters in this study, the cavitation threshold is more sensitive to the change of frequency.

## Highlights

• Performing numerical simulations of the cavitation threshold by solving the dynamic equations of the multi-bubble model with different radii.
• The cavitation threshold *P* and frequency *f* can be expressed as *P* = A*f*^α^ *+* B.
• As the number of bubbles increases, the cavitation threshold indicates a non-monotonic trend.
• The cavitation threshold in viscoelastic medium is not consistently higher than that in water.

## 1 Introduction

Acoustic cavitation refers to the formation and subsequent dynamic behavior of bubbles when a liquid suffers from a sufficient pressure drop [1]. It is highly valuable across numerous domains, including biomedical engineering [[2], [3], [4]], food processing [5,6], petrochemical industry [7,8], environmental treatment [9,10] and chemical reactions catalysis [[11], [12], [13]]. In medical science, microbubbles not only serve as contrast agents to enhance the imaging quality and diagnostic efficiency [14], but also play a crucial role in tumor ablation treatment [15]. However, cavitation may also lead to mechanical or thermal damage to healthy biological tissues surrounding the treatment target. Therefore, precise control of ultrasonic parameters is essential to minimize the risk of unnecessary damage. The acoustic cavitation threshold refers to the minimum sound pressure amplitude required to induce cavitation in a liquid under defined conditions [16]. A comprehensive understanding and precise control of the cavitation threshold are crucial for ensuring the safety of therapeutic ultrasound treatments and for developing more effective therapeutic methods. Several different criteria are used to determine the onset of cavitation, including those based on bubble radius (e.g., *R*_max_ ≥ 2*R*_0_ [17], where *R*_max_ and *R*_0_ are the maximum radius during the bubble vibration and the initial radius of the bubble), bubble wall velocity (e.g., *U*_max_ ≥ *c_l_* [18] and *U*_max_ ≥ *c_g_* [19], with *U*_max_ denoting the maximum vibration velocity of the bubble wall during vibration, *c_l_* and *c_g_* being the sound velocities of the liquid outside the bubble and the gas inside the bubble, respectively), temperature inside the bubble (e.g., *T*_max_ ≥ 1550 K [20] and *T*_max_ ≥ 5000 K [21], where *T*_max_ is the maximum temperature inside the bubble during the vibration), as well as criteria based on subharmonic components [22] and the broadband noise [23] in the cavitation noise spectrum. The cavitation thresholds derived from these diverse criteria exhibit variations.

Three general types of methods can be used in the research of the cavitation threshold, i.e., theoretical approaches [16], experimental investigations [[24], [25], [26]], and numerical simulations [[27], [28], [29]]. The classical Blake cavitation threshold theory provides a certain degree of insight into the difficulty of nucleated cavitation; however, it neglects the impact of frequency, thereby diminishing the accuracy of its predictions in the high-frequency range (several megahertz) [30]. Since the theory of bubble dynamics can provide a reference for the motion of bubbles in the cavitation field, it can also be used for the numerical calculation of the cavitation threshold. In this regard, Sponer [20] used the Keller equation to simulate nonlinear oscillations of a single bubble in water, and found that the cavitation threshold increases with viscosity, especially in the high-frequency (10 MHz) region, and an increase in surface tension also raises the cavitation threshold. Hong et al. [31] employed the Rayleigh-Plesset and Keller-Miksis equations to simulate the bubble dynamics behaviors, incorporating the energy equations for both the bubble and liquid regions along with the phase transition rate. This approach allowed them to develop a numerical model for determining the cavitation threshold of a single bubble in water. The numerical calculation results show that phase change vapor has significant influences on the bubble dynamics during acoustic cavitation. With the increase of bubble size and frequency, the cavitation threshold will exceed the Black threshold. Yang et al. [32] developed a single-bubble dynamics model in a viscoelastic medium and predicted the cavitation threshold using the *R*_max_ ≥ 2*R*_0_ criterion, and they found that the presence of elasticity increased the threshold of inertial cavitation. Wang et al. [33] numerically solved the dynamics equation of a single bubble in viscoelastic medium and investigated the effect of dual-frequency excitation on the cavitation threshold by using the *R*_max_ ≥ 2*R*_0_ criterion, and their findings revealed that introducing an additional acoustic wave frequency significantly reduced the inertial cavitation threshold. Specifically, the cavitation threshold was found to be lower when the two frequency components had the same amplitude, a phase difference of 0, and a larger frequency difference. Suo et al. [34] used a single-bubble model to numerically simulate the cavitation threshold in water and viscoelastic medium under dual-frequency and triple-frequency ultrasonic excitation. They selected the judgment criteria of the cavitation threshold as *R*_max_ ≥ 2*R*_0_ and *U*_max_ ≥ *c_l_*, and concluded that on the basis of the dual-frequency excitation, the introduction of a third frequency component can further reduce the cavitation threshold, but the effect of its enhancement is relatively small, and the combination effect may be weaker than single-frequency excitation if there is a multiplicative relationship between two frequencies. Some scholars further used GPU accelerated computation to analyze the effect of dual-frequency ultrasound on cavitation thresholds [35,36]. Qin et al. [37] established the envelope double-bubble model in viscoelastic medium and performed numerical calculations to determine the cavitation threshold by *R*_max_ ≥ 2*R*_0_ criterion. They found that the inertial cavitation threshold decreases as bubble surface tension increases, and rises with an increase in shell viscoelasticity. Furthermore, as the distance between bubbles decreases or the initial radius of larger bubbles increases, the inertial cavitation threshold progressively rises.

The research on cavitation threshold faces many challenges in practical applications. In therapeutic ultrasound, high-frequency signals are predominantly used. However, the classical Blake cavitation threshold theory does not account for frequency effects, leading to inaccuracies in high-frequency scenarios. Compared with low-frequency signals, the attenuation of high-frequency signals is more rapid, this characteristic may lead to the loss of effective information, affect the accuracy of cavitation event detection, and further affect the judgment of cavitation threshold. In addition, high-frequency ultrasound is often accompanied by the coupling of thermal effect and mechanical effect. The heat generated by the cavitation effect may change the local characteristics of the medium, thereby affecting the cavitation threshold. Furthermore, the biological tissue is a kind of viscoelastic medium with complex constitutive relations [38]. The growth and collapse processes of cavitation bubbles in it are regulated by viscosity, shear modulus, relaxation time, etc., and their dynamic behaviors are different from those in Newtonian fluids. These nonlinear responses make the traditional bubble dynamics model based on Newtonian fluids can not be directly applied to the determination of the cavitation threshold of biological tissue.

In the field of therapeutic ultrasound, ultrasonic cavitation typically occurs as bubble clusters within biological tissues. The interactions among bubbles, as well as the viscoelasticity of the medium, significantly influence the bubble dynamics behavior [[39], [40], [41], [42], [43], [44], [45]]. These factors, in turn, impact the calculation of the cavitation threshold. So far, there have been no numerical calculations of the cavitation threshold for bubble clusters containing a large number of bubbles of different sizes, and no quantitative relationship between the cavitation threshold and frequency in such case either. To fill in this gap, this study employed numerical simulations to investigate the variations in the cavitation threshold in water and viscoelastic media by using a previously bubble cluster dynamics model we have previously established [46]. Then we gave the empirical equations of cavitation threshold versus frequency under different criteria. The effects of different cavitation threshold criteria, bubble dynamics models, the number of bubbles and the medium's viscoelasticity on the cavitation threshold were systematically analyzed, and also compared the calculation results with the experimental data of previous researchers. The work aims to enhance our understanding and predictive capabilities regarding ultrasonic cavitation phenomena, thereby providing more accurate theoretical guidance for its applications in ultrasound therapy and industrial processes. Not only will this study help optimize the application effects of ultrasound technology, but it may also offer novel perspectives and methodologies for future research on the cavitation threshold.

## 2 Theoretical foundation

In this study, we employ the bubble cluster dynamics model we previously developed [46], which is grounded on the Keller-Miksis bubble dynamics equations and the Kelvin-Voigt viscoelastic constitutive relationship. The Kelvin-Voigt model is chosen here because of the simpler form of its constitutive equation and the fact that there do exist some properties of biological tissues that can be described by it (such as the liver) [47]. In addition, this model also provides some experimental data [48], which offers the possibility of comparing theoretical calculation results with experimental data under certain conditions.

This model enables us to characterize the vibration behavior of a bubble cluster containing a large number of bubbles of varying sizes in a viscoelastic medium.

The number of types of bubble radius in the bubble cluster is *q*, and the initial radius for the bubble of each radius is *R*_0_*_i_*, the instantaneous radius is *R_i_*(*t*) (*i* = 1,2,…,*q*). The vibration of a bubble of the *i*-th (*i* = 1,2,…,*q*) size can be described by the following equation:

(1) $$\left(1-\frac{{\dot{R}}_{i}}{c_{l}}\right)R_{i}{\ddot{R}}_{i}+\frac{3}{2}{\dot{R}}_{i}^{2}\left(1-\frac{{\dot{R}}_{i}}{3c_{l}}\right)=\left(1+\frac{{\dot{R}}_{i}}{c_{l}}\right)\frac{p_{i}}{\rho }+\frac{R_{i}}{\rho c_{l}}\frac{dp_{i}}{\mathrm{dt}}-\sum_{j=1}^{q}S_{j}\left(2R_{j}{\dot{R}}_{j}^{2}+R_{j}^{2}{\ddot{R}}_{j}\right)$$

where

(2) $$p_{i}=\left(p_{0}-p_{v}+\frac{2\sigma }{R_{0i}}\right){\left(\frac{R_{0i}}{R_{i}}\right)}^{3\kappa }-p_{0}+p_{v}-\frac{2\sigma }{R_{i}}-\frac{4\mu }{R_{i}}{\dot{R}}_{i}-\frac{4G}{3R_{i}^{3}}\left(R_{i}^{3}-R_{0i}^{3}\right)-p_{s}\left(t\right)$$

*ρ* is the density of the liquid outside the bubble, *R* is the instantaneous radius of the bubble, the addition of · above represents the derivative of time, *c_l_* is the sound velocity in the medium, *p*_0_ is the ambient static pressure, *p_v_* is the vapor pressure inside the bubble, *σ* is the surface tension coefficient of the bubble, *κ* is the polytropic index of the gas, μ and G are the viscosity and shear modulus of the medium, respectively, *p_s_*(*t*) is the instantaneous driving sound pressure:

(3) $$p_{s}\left(t\right)=-p_{a}\sin\left(2\pi ft\right)$$

The model assumes that all the bubbles of the same size are uniformly distributed and vibrate, so

(4) $$S_{i}=\sum_{k=1}^{N_{i}}\frac{1}{r_{\mathrm{ik}}}\approx \int_{0}^{\delta r_{i}}\frac{1}{r^{\prime }}4\pi n_{i}r^{\prime 2}dr^{\prime }=2\pi n_{i}\delta r_{i}^{2}$$

*r_ik_* (*i* = 1,2,…,*q*; *k* = 1,2,…,*N_i_*) represents the distance between the center of the *k*-th bubble among all (*N_i_* bubbles) of the *i*-th size and the currently solved bubble. *δr_i_* (*i* = 1,2,3…, *q*) is the distance considered for the bubble–bubble interactions of the *i*-th type, which can be taken as a value related to the bubble number density (*n_i_*) − the average bubble distance [49,50]:

(5) $$\delta r_{i}=\frac{1}{{\left(n_{i}\right)}^{1/3}}$$

Substituting Eq. (5) into Eq. (4), we can get

(6) $$S_{i}=2\pi n_{i}^{1/3}$$

Details regarding the model establishment can be referred to [46].

Our assumption is that the gas within the nucleus behaves as an ideal gas and the transition from the initial state to a radius *R*(*t*) is an adiabatic process. Therefore, the temperature within the bubble can be determined by the following equation:

(7) $$T=T_{0}{\left(\frac{V_{0}}{V}\right)}^{\kappa -1}=T_{0}{\left(\frac{R_{0}}{R}\right)}^{3\left(\kappa -1\right)}$$

*T*_0_ is the initial temperature inside the bubble, and here *κ* is the adiabatic coefficient of the gas.

## 3 Numerical calculations and analysis

The bubble dynamics equation is solved numerically by using the adaptive four-fifth-order Runge-Kutta algorithm with variable step size. The target equation Eq. (1) (a system of differential equations containing *q* second-order differential equations) is written in matrix form to facilitate its solution. The solution to the equation is the bubble radius function *R*(*t*) and bubble wall velocity *U*(*t*). Subsequently, *R*(*t*) is substituted into Eq. (7) to calculate the temperature *T*. In the following numerical calculations, liver is selected as the specific viscoelastic medium, with its parameter values sourced from the literature [36]. Unless otherwise specified, the values of parameters are shown in Table 1, where 'Number of bubble radius types' is for the Multi-bubble with different radii model. In this model, as long as cavitation occurs in a bubble of any size, it is considered that the excitation sound pressure amplitude has reached the cavitation threshold (This statement is explained in detail in Section 3.1). In this study, 60 cycles were calculated for the solution of Eq. (1). To avoid the influence of transient processes, the data within the 35th to 55th cycle were selected to determine the cavitation threshold. Unless otherwise specified, it is considered that the number of bubbles of each size is the same, which is *Number*/*q*.

**Table 1:** Parameter values in numerical calculations.

	Water	Viscoelastic medium
Surface tension coefficient *σ* (N/m)	0.0725	0.056
Sound velocity *c_l_* (m/s)	1500	1549
Density *ρ* (kg/m^3^)	998	1060
Viscosity μ (mPa·s)	1	9
Shear modulus G (kPa)	0	40
Static pressure *p*_0_ (kPa)	101.3	
Vapor pressure *p_v_* (kPa)	2.33	
Adiabatic coefficient *κ*	1.4	
Initial temperature inside the bubble T_0_ (K)	290	
Sound velocity of the gas inside bubbles *c_g_* (m/s)	340	
Initial radius *R*_0_ (μm)	0.5, 1, 3, 6, 8, 10	
Number of bubble radius types *q*	6	
Volume of the bubble cloud *V* (m^3^)	2.7 × 10^-8 *^	

*: The bubble cloud is a cube with a side length of 3 mm.

Attention:

The following conditions need to be met when using the model for numerical calculations:

a. The number of bubbles in the studied area is sufficient so that Eqs. (5) and (6) can be established;

b. To avoid the shielding effect of bubbles, the ratio of the total volume of bubbles to the volume of the study area does not exceed 5 %;

c. To satisfy the condition of simultaneous vibration of bubbles of each size, the average bubble distance *δr_i_* is not more than one eighth of the wavelength, i.e., *δr_i_* < λ/8.

### 3.1 Comparison of cavitation thresholds under different criteria (In water and viscoelastic medium)

**Fig. 1:**
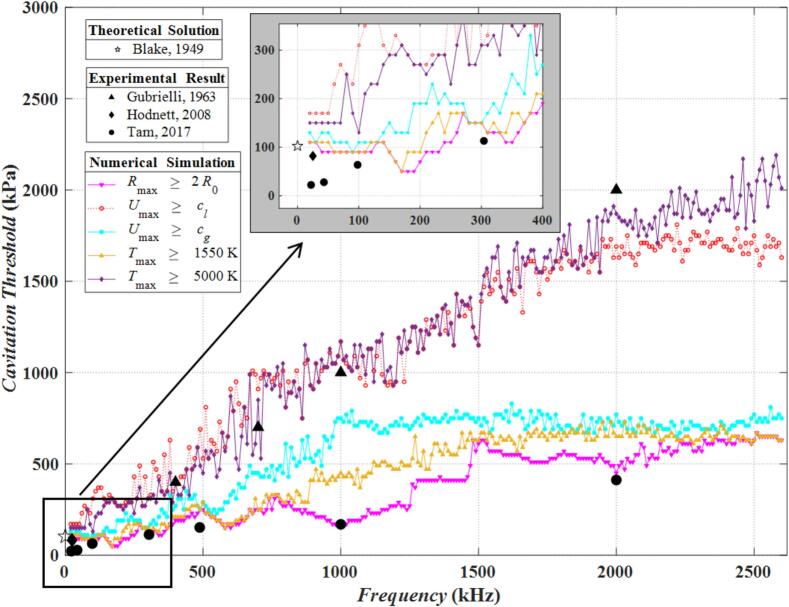
Cavitation threshold versus frequency for five criteria calculated by the Multi-bubble with different radii model (in water).

**Fig. 2:**
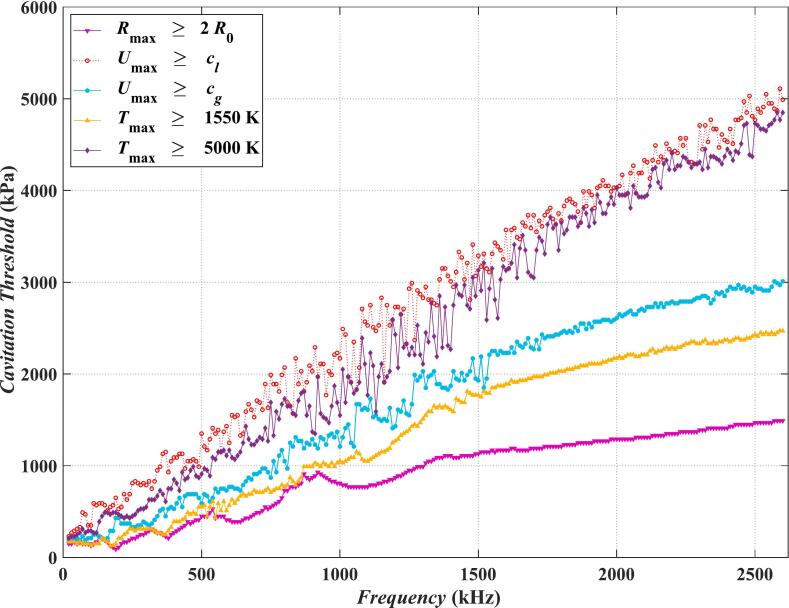
Cavitation threshold versus frequency for five criteria calculated by the Multi-bubble with different radii model (in viscoelastic medium).

Fig. 1 and Fig. 2 show the relationship between the cavitation threshold and the frequency in water and viscoelastic medium calculated by the Multi-bubble with different radii model under five criteria. Here the number of bubbles '*Number*' is 4 × 10^5^. We also calculated the value of the Blake cavitation threshold. As the larger the initial radius of the bubble, the lower the Blake cavitation threshold. In the bubble cluster, the Blake cavitation threshold of the largest bubble (*R*_0_ = 10 μm) represents that of this bubble cluster, which is 102.5 kPa. Since it does not reflect the relationship with frequency, the value of the Blake cavitation threshold is marked with a hollow pentagram at a horizontal coordinate (frequency) of 0. Some previous experimental results [[51], [52], [53]] are also labeled in Fig. 1. The image of the frequency range 0–400 kHz indicated by the arrow is enlarged and displayed with a gray background in the upper middle part of Fig. 1. It can be seen that the Blake cavitation threshold is closer to the numerical calculation curves at low frequencies (about 20 kHz), and its difference between the numerical calculation curves and experimental results gradually increases as the frequency rises.

The calculation reveals that the curves in Fig. 1 and Fig. 2 can be expressed in the form of *P* = A*f* ^α^ *+* B, where *P* (in Pa) represents the cavitation threshold, *f* (in Hz) represents the frequency, A, B and α depend on the medium properties and different criteria of the cavitation threshold. Specifically, writing the cavitation thresholds determined by *R*_max_ ≥ 2*R*_0_, *U*_max_ ≥ *c_g_*, *U*_max_ ≥ *c_l_*, *T*_max_ ≥ 1550 K and *T*_max_ ≥ 5000 K as *P_R_*, *P_cg_*, *P_cl_*, *P_T_*_1_, *P_T_*_2_, respectively. The values of A, B and α can be obtained by fitting the curves in Fig. 1 and Fig. 2, as shown in Table 2. In water, the value of constant term B of the criteria *U*_max_ ≥ *c_g_* is 9 × 10^4^, which is closest to the Blake cavitation threshold. It can be found that with the exception of *P_T1_* and *P_T_*_2_ in viscoelastic medium, the exponential term α takes a value between 0.5 and 1. α in viscoelastic medium is generally greater than that in water.

**Table 2:** Values of A, B and α in the fitted curves for cavitation threshold versus frequency (*P* = A*f*^α^ *+* B) in water and viscoelastic medium.

		**A**	**α**	**B**
**Water**	*P_R_*	0.36	0.97	5 × 10^4^
	*P_T_*_1_	18.03	0.72	5 × 10^4^
	*P_cg_*	244.6	0.54	9 × 10^4^
	*P_cl_*	28.68	0.75	1.7 × 10^5^
	*P_T_*_2_	4.10	0.89	1.3 × 10^5^
**Viscoelastic medium**	*P_R_*	7.58	0.83	9 × 10^4^
	*P_T_*_1_	0.70	1.02	1.3 × 10^5^
	*P_cg_*	1.38	0.99	1.9 × 10^5^
	*P_cl_*	10.85	0.88	2.3 × 10^5^
	*P_T_*_2_	0.47	1.09	2.1 × 10^5^

As shown in Fig. 1 and Fig. 2, due to the existence of viscoelasticity, the cavitation threshold in the viscoelastic medium is generally higher than that in water at the same frequency, and the cavitation threshold curves in the viscoelastic medium are smoother than those in water (more obvious above 1000 kHz). No matter it is in water or in the viscoelastic medium, the cavitation threshold with the increase of the frequency shows an upward trend. Because both the acoustic oscillation period and the duration of the rarefaction phase are shortened with the increase of frequency, it is not easy to generate cavitation bubbles [54] and to accumulate energy in the cavitation bubbles. Therefore, in order to generate acoustic cavitation at high frequencies, there must be a higher sound pressure.

The highest cavitation thresholds are determined by *T*_max_ ≥ 5000 K and *U*_max_ ≥ *c_l_* criteria, followed by *U*_max_ ≥ *c_g_*, *T*_max_ ≥ 1550 K and *R*_max_ ≥ 2*R*_0_ in that order. In water, the cavitation thresholds determined by *T*_max_ ≥ 5000 K can reach 2000 kPa at a frequency of 2500 kHz, whereas that determined by *R*_max_ ≥ 2*R*_0_ is only 600 kPa. In viscoelastic medium, the cavitation threshold determined by *T*_max_ ≥ 5000 K can reach 4800 kPa, while that determined by *R*_max_ ≥ 2*R*_0_ is only 1500 kPa. It can be found that there is a big difference between the cavitation thresholds calculated by different criteria. Only by calculating the cavitation threshold in accordance with the actual conditions can one obtain results with practical implication. For example, when used for treatment, the *R*_max_ ≥ 2*R*_0_ criterion is the most appropriate criterion due to the lower cavitation threshold calculated from the safety point of view. In water, the experimental results of Gubrielli et al. [51] in 1963 (Fig. 1 solid black triangle markers) are closer to the purple (*T*_max_ ≥ 5000 K) and red (*U*_max_ ≥ *c_l_*) curves. The experimental results of Hodnett et al. in 2008 [52] (Fig. 1 solid black diamond marker) is closer to the pink (*R*_max_ ≥ 2*R*_0_) and the yellow curve (*T*_max_ ≥ 1550 K) at low frequencies. The experimental results of Tam et al. [53] in 2017 (Fig. 1 solid black circle markers) are closer to the pink curve (*R*_max_ ≥ 2*R*_0_).

**Fig. 3:**
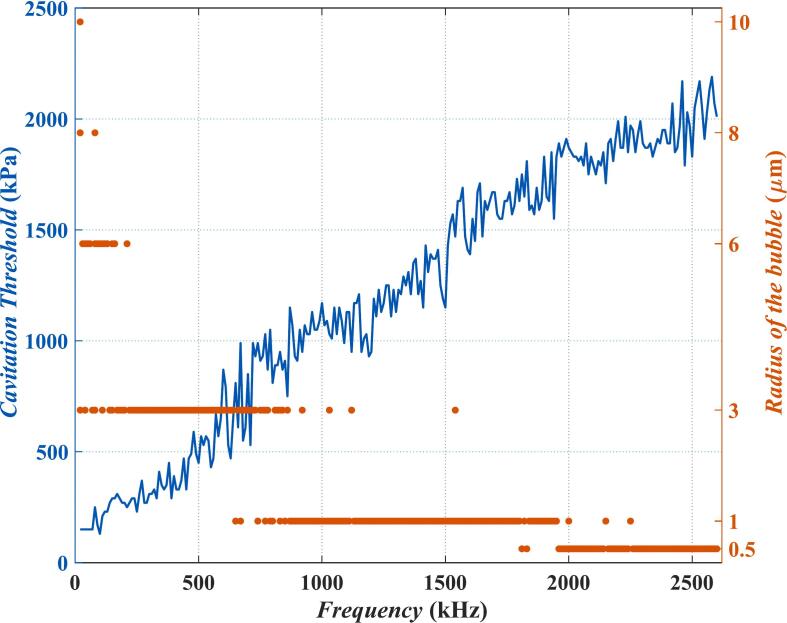
The cavitation threshold and the corresponding bubble radius calculated by the *T*_max_ ≥ 5000 K criterion (in water).

As mentioned before, since bubbles in a bubble cluster vary in size, the cavitation threshold of a bubble cluster is calculated as follows: as long as bubbles of any size reach the cavitation condition, the current excitation sound pressure is regarded as the cavitation threshold of the bubble cluster. The following example is given to explain this point. Fig. 3 has two y-axes on the left and right. The values of the blue curve are marked by the left y-axis, which represents the variation of the cavitation threshold with frequency in water calculated by the criterion of *T*_max_ ≥ 5000 K (i.e., the purple curve in Fig. 1). The red solid circles are calibrated by the right y-axis, which represents the size of bubbles undergoing cavitation at the current frequency. As is shown in Fig. 3, when the frequency is lower than 300 kHz, only the bubbles with *R*_0_ = 10 μm, 8 μm, 6 μm and 3 μm can reach the cavitation threshold. As the frequency rises, generally only the bubbles with *R*_0_ = 3 μm can reach the cavitation threshold in the frequency band of 1000 kHz-1800 kHz. As the frequency rises above 2000 kHz, only bubbles with *R*_0_ = 0.5 μm can reach the cavitation threshold, and the cavitation threshold is very high at this point (close to 2000 kPa). In summary, the larger bubbles in the cluster reach the cavitation threshold first at lower frequencies. As frequency increases, the size of the bubbles that first cavitate decreases and the cavitation threshold rises. At low frequencies, Blake's cavitation threshold theory indicates that smaller bubbles have higher cavitation thresholds. Therefore, the first to cavitate are the large-sized bubbles in the bubble cluster. As frequency increases, the difference between large bubbles' resonance frequency and the current frequency becomes greater. Consequently, these large bubbles have low amplitudes and rarely cavitate. At this time, the small bubbles in the cluster are the most likely to reach the cavitation threshold. Due to the reason mentioned before (higher frequency means less energy accumulation per cycle for bubbles), the value of the cavitation threshold at this time is very high.

**Fig. 4:**
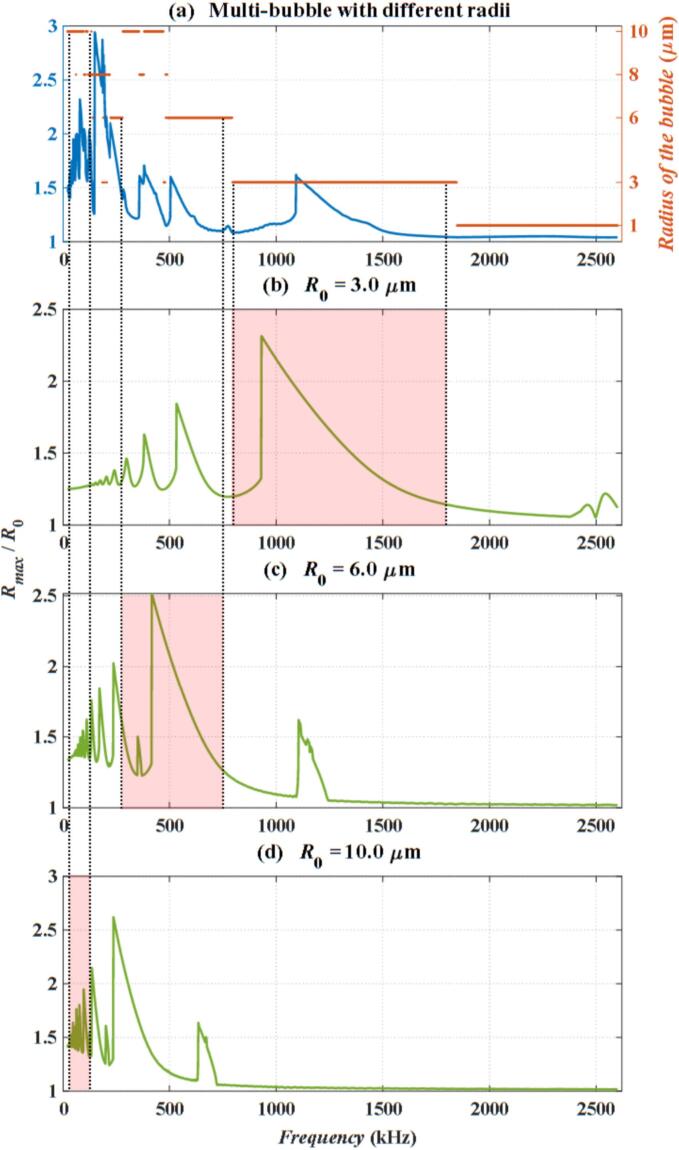
The frequency response curve. (a)Multi-bubble with different radii; (b) Single bubble (*R*_0_ = 3 μm); (c) Single bubble (*R*_0_ = 6 μm); (d) Single bubble (*R*_0_ = 10 μm).

To gain a better understanding of the vibrational characteristics of bubbles of varying sizes within a bubble cluster, we plot the frequency response curve, which illustrates the maximum expansion ratio (*R*_max_ / *R*_0_), defined as the ratio of the maximum radius to the initial radius of the bubble, as a function of frequency. In the case of Multi-bubble with different radii, at a given frequency, the maximum value of *R* / *R*_0_ among all bubbles, regardless of size, is chosen as the representative maximum expansion ratio for that frequency. The corresponding calculation results are shown in Fig. 4.

Fig. 4(a) shows the frequency response curve for the Multi-bubble with different radii model, while Fig. 4(b), Fig. 4(c), Fig. 4(d) present the frequency response curves for the Single bubble model at *R*_0_ = 3 μm, 6 μm and 10 μm, respectively. In Fig. 4(a), the blue curve (scaled by the left y-axis) represents the frequency response curve, whereas the red solid circles (scaled by the right y-axis) indicate the bubble size that exhibits the maximum expansion ratio at each frequency. Due to their high density, these circles appear as continuous horizontal red solid and dashed lines in the figure. The size of bubble with the maximum response within the bubble cluster varies across different frequency ranges. Below 100 kHz, bubbles with *R*_0_ = 10 μm exhibit the maximum expansion ratio, thus determining the frequency response curve within this frequency range. As the frequency increases to 250kH −750 kHz, bubbles with *R*_0_ = 6 μm display the maximum expansion ratio, characterized by a large amplitude and an obvious response. Consequently, the frequency response curve in this range is based on the maximum expansion ratio of bubbles with *R*_0_ = 6 μm. Similarly, within the 800 kHz-1700 kHz range, bubbles with *R*_0_ = 3 μm become dominant, while at frequencies exceeding 1800 kHz, smaller bubbles with *R*_0_ = 1 μm exhibit the most substantial response. In each frequency band, the dominant role in the bubble cluster is played by bubbles of different sizes. The red-shaded regions in Fig. 4(b), Fig. 4(c), and Fig. 4(d) correspond to the frequency bands in which the respective bubbles play a dominant role, as indicated in Fig. 4(a).

### 3.2 Comparison of cavitation thresholds in different bubble dynamics models (in water)

Following the analysis of the vibration and cavitation characteristics of the Multi-bubble with different radii model, this section presents a comparative study with other bubble dynamics models. Specifically, three models are considered, namely, the previously discussed Multi-bubble with different radii, a Multi-bubble with the same radius, and a Single bubble model. From the preceding subsection, it was established that the cavitation threshold determined by *R*_max_ ≥ 2*R*_0_ criterion is the lowest among the evaluated thresholds. Since safety is an important factor in ultrasound treatment, the *R*_max_ ≥ 2*R*_0_ criterion is adopted for all the following calculations of cavitation threshold to ensure a conservative approach in treatment applications.

**Fig. 5:**
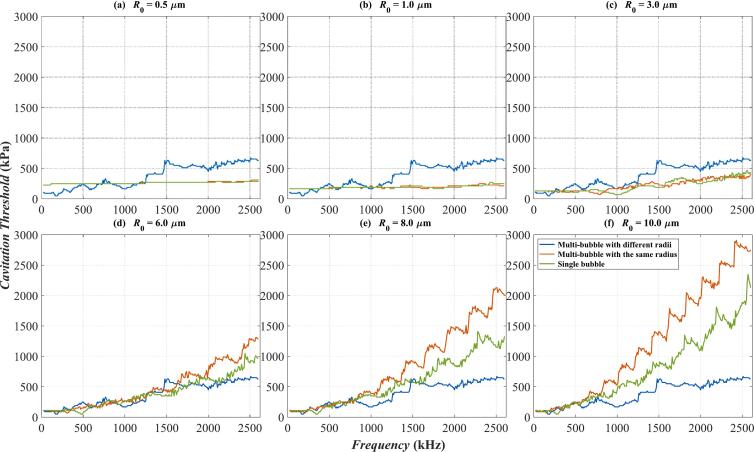
Variation of cavitation thresholds with frequency calculated by three models in water. (a) *R*_0_ = 0.5 μm; (b) *R*_0_ = 1 μm; (c) *R*_0_ = 3 μm; (d) *R*_0_ = 6 μm; (e) *R*_0_ = 8 μm; (f) *R*_0_ = 10 μm; The blue curve: Multi-bubble with different radii. The red curve: Multi-bubble with the same radius. The green curve: Single bubble.

Fig. 5 presents the cavitation threshold as a function of frequency, calculated based on the *R*_max_ ≥ 2*R*_0_ criterion in water. The horizontal axis represents frequency, while the vertical axis denotes the cavitation threshold. The curves in different colors correspond to different bubble dynamics models; the blue one represents the Multi-bubble with different radii model, the red one corresponds to Multi-bubble with the same radius model, and the green one represents the Single bubble case. Notably, the blue curves remain the same across all six subfigures of Fig. 5. This consistency arises from the fact that the bubble cluster represented by the blue curves encompasses all bubble sizes within the same cluster, leading to a unique cavitation threshold. Consequently, the blue curves serve as a reference. When the bubble size is small (*R*_0_ = 0.5 μm and *R*_0_ = 1 μm represented by Fig. 5(a) and Fig. 5(b)), the red and green curves exhibit minimal fluctuation. This indicates that the cavitation threshold computed by the Multi-bubble with the same radius and Single bubble models do not change significantly with frequency in this range. Furthermore, for frequencies above 1300 kHz, the amplitudes of the red and green curves are lower than the blue curves. As the bubble radius increases (i.e., Fig. 5(a)-(f)), the amplitudes of the red and green curves computed by Multi-bubble with the same radius and Single bubble models increase accordingly. Finally, in the case of *R*_0_ = 10 μm represented by Fig. 5(f), the amplitude of the red curve is greater than that of the green one, whereas the blue curve maintains the lowest amplitude, particularly at high frequencies (above 1000 kHz). Above all, different bubble dynamics models significantly influence the value of cavitation threshold. In practical applications, the selection of an appropriate bubble dynamics model depends on the specific requirements and the primary objectives of the study.

### 3.3 Comparison of cavitation thresholds in water and viscoelastic medium

The preceding subsection analyzed and compared the effect of different bubble dynamics models on the cavitation threshold. In this section our focus is to examine how variations in medium type influence the cavitation threshold.

**Fig. 6:**
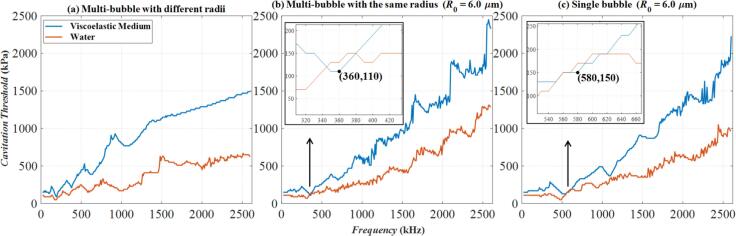
The relationship of cavitation threshold with frequency calculated by three models in water and viscoelastic medium. (a)Multi-bubble with different radii; (b) Multi-bubble with the same radius (*R*_0_ = 6 μm); (c) Single bubble (*R*_0_ = 6 μm); The blue curve: Viscoelastic medium. The red curve: Water.

**Fig. 7:**
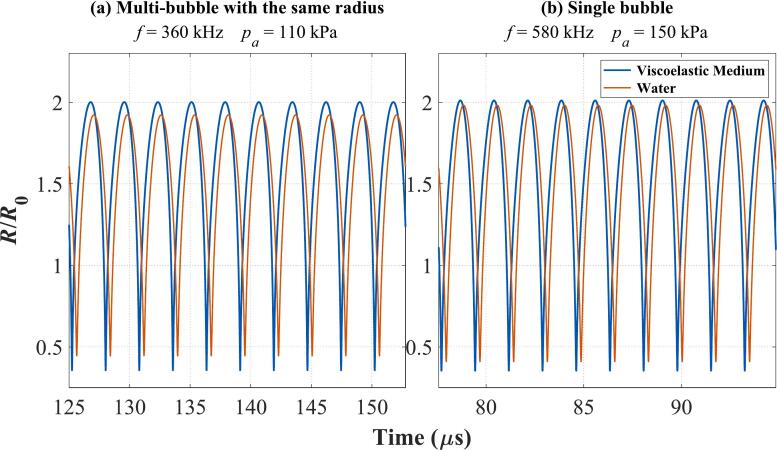
Bubble radius versus time curves for the two points labeled in Fig. 4(b) and Fig. 4(c). (a)Multi-bubble with the same radius (*R*_0_ = 6 μm) for *f* = 360 kHz, *p_a_* = 110 kPa; (b) Single bubble (*R*_0_ = 6 μm) for *f* = 580 kHz, *p_a_* = 150 kPa; The blue curve: Viscoelastic medium. The red curve: Water.

Fig. 6 illustrates the frequency variation of cavitation thresholds calculated by *R*_max_ ≥ 2*R*_0_ under three models in water and viscoelastic medium. Specifically, Fig. 6(a) presents results for a Multi-bubble with different radii model while Fig. 6(b) and Fig. 6(c) depict the models of Multi-bubble with the same radius and Single bubble, respectively, both with an initial radius of *R*_0_ = 6 μm. Across all models, the red curve remains consistently lower than the blue curve over most frequency ranges. This indicates that the presence of viscoelasticity results in a higher cavitation threshold compared to water under the same conditions. This trend aligns with our general expectations. However, two exceptions are observed: at approximately 360 kHz in Fig. 6(b) and 580 kHz in Fig. 6(c), the red and blue curves overlap. These two positions are marked with arrows and magnified (the small black-framed figures in Fig. 6(b) and Fig. 6(c)). These points, highlighted with arrows and magnified in the inset figures, reveal that at these specific frequencies, the red curve surpasses the blue curve in amplitude, indicating that the cavitation threshold in water is higher than that in the viscoelastic medium. Fig. 7 further illustrates these points by presenting the corresponding bubble radius variations. Fig. 7(a) corresponds to the case of *f* = 360 kHz, *p_a_* = 110 kPa in Fig. 6(b), and Fig. 7(b) corresponds to the case of *f* = 580 kHz, *p_a_* = 150 kPa in Fig. 6(c). In both cases of Fig. 7(a) and Fig. 7(b), the amplitude of the blue curve exceeds that of the red curve, indicating that the normalized bubble radius represented by the blue curve has reached 2. This confirms that under the same excitation sound pressure and frequency, cavitation occurs in the viscoelastic medium, but not in water. This phenomenon is observed in both the Multi-bubble with the same radius and Single bubble model. Due to the involvement of nonlinear effects, bubble responses, and other complex factors, it is challenging to generalize that the cavitation threshold in the viscoelastic medium is always higher than that in water under identical excitation conditions.

**Fig. 8:**
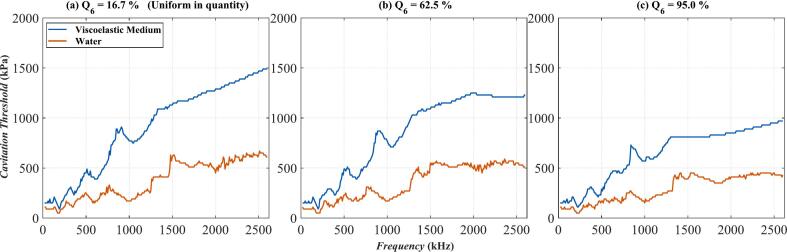
The relationship of cavitation threshold with frequency when the proportion of bubbles of *R*_0_ = 6 μm in the total number of bubbles Q_6_ is different. (a) Q_6_ = 16.7 %; (b) Q_6_ = 62.5 %; (c) Q_6_ = 95.0 %;The blue curve: Viscoelastic medium. The red curve: Water.

By observing and comparing the Multi-bubble with different radii model (Fig. 6(a)) with the Multi-bubble with the same radius model (Fig. 6(b)), we raise a question: In the extreme case of Fig. 6(a) − when the number of *R*_0_ = 6 μm bubbles is much greater than that of bubbles of other sizes, will the two curves in Fig. 6(b) intersect, that is, the cavitation threshold in the viscoelastic medium is lower than that in water? To check this issue, some additional numerical calculations were done. In the Multi-bubble with different radii model, change the number of bubbles of *R*_0_ = 6 μm, define the number of *R*_0_ = 6 μm bubbles as a percentage of the total number of bubbles as Q_6_, keep the total number of bubbles constant at 4 × 10^5^, change the value of Q_6_. Except for *R*_0_ = 6 μm bubbles, the number of bubbles of other sizes is the same. The calculation results of the cavitation threshold are shown in Fig. 8. In Fig. 8(a), the number of bubbles with *R*_0_ = 6 μm is 6.67 × 10^4^, accounting for 16.7 % of the total number of bubbles, this is also a uniform distribution of the number of bubbles of each size. In Fig. 8(b) and Fig. 8(c), the number of bubbles with *R*_0_ = 6 μm is 2.5 × 10^5^ and 3.8 × 10^5^ respectively, accounting for 62.5 % and 95.0 % of the total number of bubbles. With the increase of Q_6_, the cavitation threshold gradually decreases. The Q_6_ = 95 % represented by Fig. 8(c) is the case that is closest to the Multi-bubble with the same radius model. However, the two curves in Fig. 8(c) do not cross. The blue curve is always higher than the red one, that is, the cavitation threshold in the viscoelastic medium is always higher than that in water. This is somewhat different from the rule reflected in Fig. 6(b). Because in the Multi-bubble with different radii model, although the number of bubbles with *R*_0_ = 6 μm is the largest, it is not necessarily the bubbles with *R*_0_ = 6 μm that undergo cavitation (i.e., the phenomenon reflected in Fig. 3). While in the Multi-bubble with the same radius model, there is only one size of bubble, *R*_0_ = 6 μm. If cavitation occurs, it must be that the bubble of this size meets the cavitation conditions. The setting of models lead to unique patterns in the calculation results of the two figures.

**Fig. 9:**
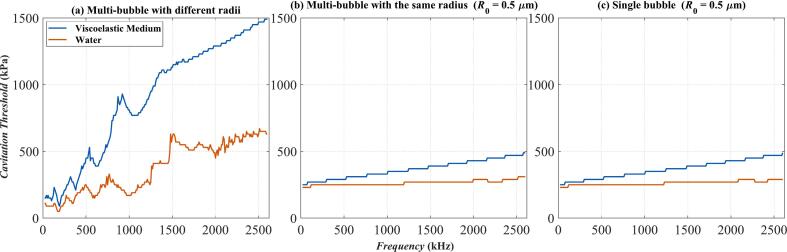
The relationship of cavitation threshold with frequency calculated by three models in water and viscoelastic medium. (a)Multi-bubble with different radii; (b) Multi-bubble with the same radius (*R*_0_ = 0.5 μm); (c) Single bubble (*R*_0_ = 0.5 μm); The blue curve: Viscoelastic medium. The red curve: Water.

Similar to Fig. 6, Fig. 9 shows the variation of cavitation thresholds with frequency in water and viscoelastic media. Fig. 9(b) and Fig. 9(c) are respectively the models of Multi-bubble with the same radius and Single bubble for *R*_0_ = 0.5 μm. It can be seen that in Fig. 9(b) and Fig. 9(c), both curves show a trend of slowly increasing with frequency, and there is no situation where the cavitation threshold in the viscoelastic medium is lower than that in water. Within the studied frequency range, the cavitation threshold in Fig. 9(b) and Fig. 9(c) changes less than 300 kPa, while in Fig. 9(a), the cavitation threshold changes nearly 1500 kPa.

**Fig. 10:**
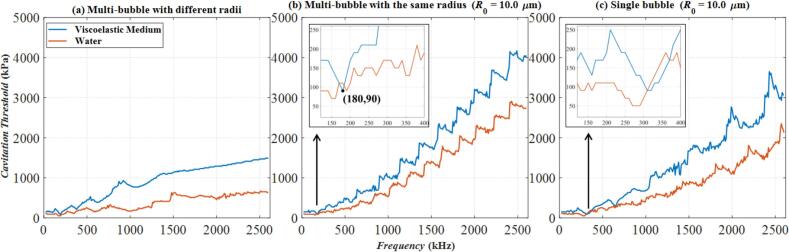
The relationship of cavitation threshold with frequency calculated by three models in water and viscoelastic medium. (a)Multi-bubble with different radii; (b) Multi-bubble with the same radius (*R*_0_ = 10 μm); (c) Single bubble (*R*_0_ = 10 μm); The blue curve: Viscoelastic medium. The red curve: Water.

Observe the bubbles with *R*_0_ = 10 μm. Fig. 10 shows the variation of the cavitation threshold with frequency in water and viscoelastic media. Fig. 10(b) and Fig. 10(c) respectively show the models of Multi-bubble with the same radius and Single bubble for *R*_0_ = 10 μm. The amplitudes of the two curves in Fig. 10(b) and Fig. 10(c) increase with the increase of frequency. Within the studied frequency range, the cavitation threshold in Fig. 10(b) and Fig. 10(c) changes approximately 3000 kPa, while in Fig. 10(a), the cavitation threshold only changes nearly 1500 kPa. From Fig. 6, Fig. 9 and Fig. 10, in the case of Multi-bubble with the same radius and Single bubble models, the larger the bubble radius, the higher the increase amplitude of its cavitation threshold with frequency. Similar to Fig. 6, the intersection of the two curves also occurs in Fig. 10(b) and Fig. 10(c). The detailed situation is magnified and marked by the black arrows. In Fig. 10(b), cavitation has occurred in the viscoelastic medium when *f* = 180 kHz, *p_a_* = 90 kPa, whereas no cavitation has occurred in the water.

**Fig. 11:**
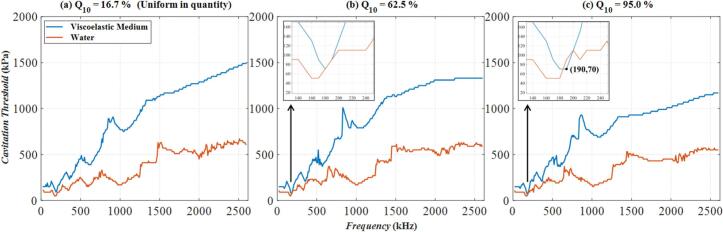
The relationship of cavitation threshold with frequency when the proportion of bubbles of *R*_0_ = 10 μm in the total number of bubbles Q_10_ is different. (a) Q_10_ = 16.7 %; (b) Q_10_ = 62.5 %; (c) Q_10_ = 95.0 %;The blue curve: Viscoelastic medium. The red curve: Water.

Using the same analytical method, in the Multi-bubble with different radii model, change the number of bubbles with *R*_0_ = 10 μm, define the percentage of the number of bubbles with *R*_0_ = 10 μm to the total number of bubbles as Q_10_, keep the total number of bubbles at 4 × 10^5^ unchanged, and change the value of Q_10_. Except for the *R*_0_ = 10 μm bubble, the number of bubbles of other sizes is the same. The calculation results of the cavitation threshold are shown in Fig. 11. Fig. 11(a) represents the situation where the number of bubbles of each size is uniformly distributed. In Fig. 11(b) and Fig. 11(c), the number of bubbles with *R*_0_ = 10 μm accounts for 62.5 % and 95.0 % of the total number of bubbles, respectively. Observe Fig. 11(a)-(c). With the increase of Q_10_, the cavitation threshold in the viscoelastic medium represented by the blue curve gradually decreases, but the decrease is not significant, while the cavitation threshold in water represented by the red curve remains basically unchanged. In Fig. 11(a), when Q_10_ = 16.7 %, the two curves do not intersect. When Q_10_ increases to 62.5 % (Fig. 11(b)), the two curves start to overlap, and Q_10_ continues to increase. When it reaches 95 % (Fig. 11(c)), the two curves intersect near *f* = 190 kHz, *p_a_* = 70 kPa. Specifically magnified at the position indicated by the black arrow, it indicates that the cavitation threshold in the viscoelastic medium at this time is lower than that in water, resulting in a situation similar to the Multi-bubble with the same radius model shown in Fig. 10(b).

**Fig. 12:**
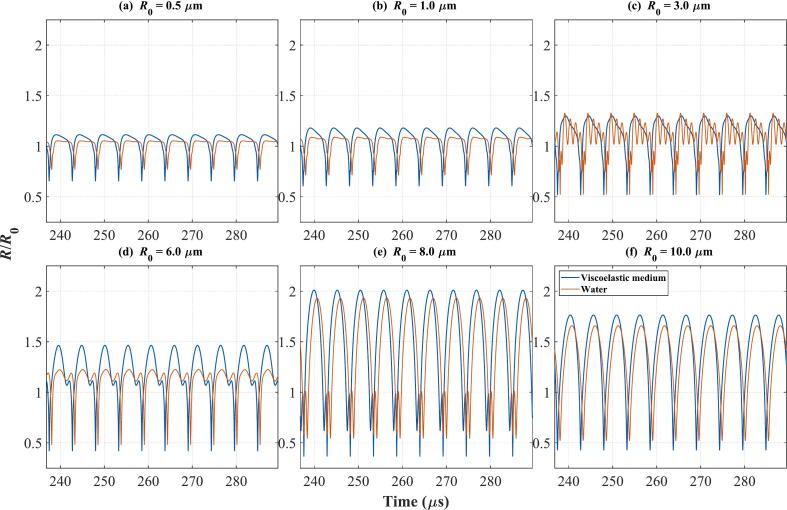
Bubble radius versus time curves for the point labeled in Fig._R 4(c) *f* = 190 kHz, *p_a_* = 70 kPa, Q_10_ = 95 %; (a) *R*_0_ = 0.5 μm; (b) *R*_0_ = 1 μm; (c) *R*_0_ = 3 μm; (d) *R*_0_ = 6 μm; (e) *R*_0_ = 8 μm; (f) *R*_0_ = 10 μm; The blue curve: Viscoelastic medium. The red curve: Water.

To observe the bubble vibration at the intersection of the two curves in Fig. 11(c), the bubble radius curves at *f* = 190 kHz, *p_a_* = 70 kPa and Q_10_ = 95 % were plotted in Fig. 12. The blue curve represents the calculation results in the viscoelastic medium, and the red curve represents that in water. It can be seen that at this time, the amplitudes of almost all sizes of bubbles in the viscoelastic medium will be greater than those in water. The bubbles with *R*_0_ = 8 μm in the viscoelastic medium (Fig. 12(e)) reach the cavitation criterion *R*_max_ ≥ 2*R*_0_, rather than the larger number of bubbles with *R*_0_ = 10 μm. Although the bubbles with *R*_0_ = 10 μm do not undergo cavitation, the change in their number is also one of the factors that promote the bubbles with *R*_0_ = 8 μm to reach the cavitation criterion. Therefore, in the cases of the Multi-bubble with different radius model (Q_10_ = 95 %) in Fig. 11(c) and the Multi-bubble with the same radius model in Fig. 10(b), although the cavitation threshold in viscoelastic media is lower than that in water in both cases, the causes of this phenomenon may not be the same.

### 3.4 The effect of viscosity and elasticity on the cavitation threshold

**Fig. 13:**
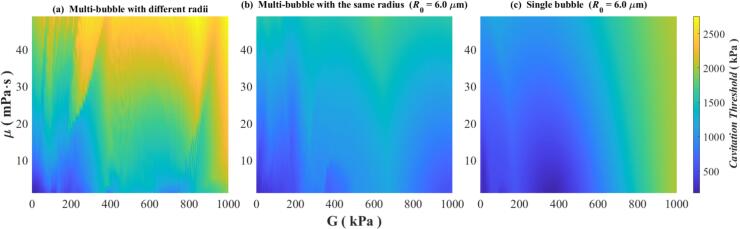
Variation of cavitation threshold with viscosity and shear modulus calculated by three models. (a) Multi-bubble with different radii; (b) Multi-bubble with the same radius (*R*_0_ = 6 μm); (c) Single bubble (*R*_0_ = 6 μm).

To further investigate the influence of the viscoelasticity of the medium on the cavitation threshold, all other parameters held constant, including the density of the medium, the speed of sound and the surface tension coefficient of the bubbles, which are assigned values corresponding to the viscoelastic medium. Only the viscosity μ and shear modulus G are varied. The cavitation threshold is determined using the criterion *R*_max_ ≥ 2*R*_0_ at a fixed frequency of *f* = 1000 kHz. The results are shown in Fig. 13, where Fig. 13(a) corresponds to a Multi-bubble with different radii, while Fig. 13(b) and Fig. 13(c) represent models of Multi-bubble with the same radius and Single bubble at *R*_0_ = 6 μm, respectively. The horizontal axis denotes the shear modulus G in unit kPa, and the vertical axis represents the coefficient of viscosity μ in unit mPa·s.

Fig. 13 illustrates that the cavitation threshold calculated by the Multi-bubble with different radii model at *f* = 1000 kHz is larger than that calculated by the Multi-bubble with the same radius and Single bubble models at *R*_0_ = 6 μm, and the cavitation threshold in Fig. 13(a) can reach more than 2500 kPa when G = 850 kPa and μ = 45 mPa·s, whereas the cavitation thresholds in Fig. 13(b) and Fig. 13(c) are only about 1470 kPa and 1850 kPa. In terms of the dependence on viscous and elastic variations, Fig. 13(a) also has a not so similar law with the other two. In the Multi-bubble with different radii model represented by Fig. 13(a), when the viscosity remains constant, the cavitation threshold exhibits a fluctuating but generally increasing trend of shear modulus. However, these fluctuations do not follow a simple pattern, indicating a more complex dependence. In the Multi-bubble with the same radius model with *R*_0_ = 6 μm represented by Fig. 13(b), cavitation threshold also presents a fluctuating trend with the increase of shear modulus, but the law is slightly simpler than that in Fig. 13(a). Notably, the maximum cavitation threshold does not occur at the highest shear modulus value (G = 1000 kPa), but rather around G = 630 kPa. The Single bubble model with *R*_0_ = 6 μm represented in Fig. 13(c) exhibits a comparatively simpler trend. In the case of low viscosity (less than 20 mPa·s), for constant viscosity, the cavitation threshold initially increases with shear modulus, then decreases, followed by another increase. The inflection points are around G = 170 kPa and G = 600 kPa. In the region of higher viscosity where μ＞20 mPa·s, the cavitation threshold increases with the increase of shear modulus, and when G = 900 kPa and μ = 45 mPa·s, the cavitation threshold can be up to 1920 kPa. Since the medium shear modulus may affect the response of the bubble cluster [46], its effect on the cavitation threshold is more complex. Despite some variations, all three subfigures in Fig. 13 generally indicate an increasing trend of the cavitation threshold with viscosity, except in Fig. 13(c) for shear modulus values exceeding 800 kPa, where the variation is minimal. Overall the effect of shear modulus (elasticity) on the cavitation threshold is larger than that of viscosity. In numerical calculations of the cavitation thresholds, the laws obtained from the Single bubble model or the Multi-bubble with the same radius model are inaccurate and differ significantly from those obtained from the Multi-bubble with different radii model. While the latter does not fully replicate real-world conditions, it provides the most accurate approximation among the three models.

### 3.5 The effect of bubble number on the cavitation threshold (in water)

**Fig. 14:**
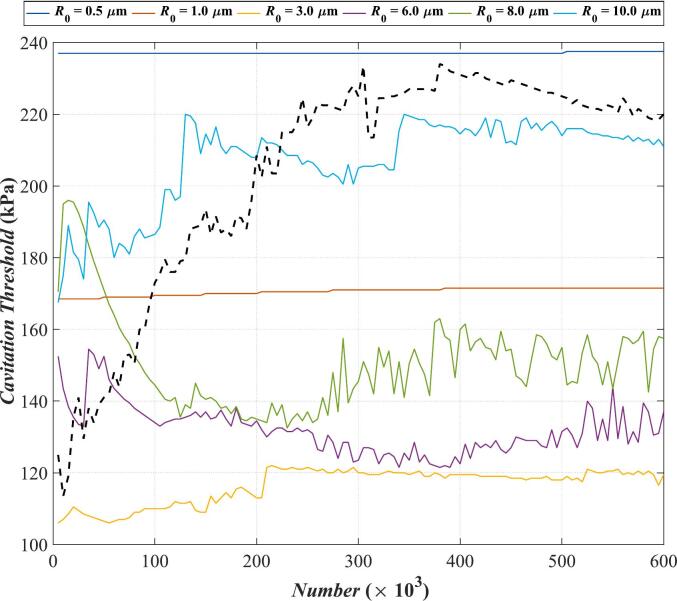
Variation of cavitation threshold in water with the number of bubbles for *f* = 500 kHz. The black dotted curve: Multi-bubble with different radii; Other colored curves: Multi-bubble with the same radius for different *R*_0_.

The preceding discussion assumes that the total number of bubbles remains constant. In this section, we analyze the variation of the cavitation threshold with the number of bubbles. In order to reduce the influence of other factors, the medium for calculation is selected as water with relatively simple properties. Fig. 14 shows the relationship between the cavitation threshold and the number of bubbles at *f* = 500 kHz, where the black dotted curve is the Multi-bubble with different radii model, and other colored curves represent the results calculated by the Multi-bubble with the same radius model for different *R*_0_.

As Fig. 14 shows, among all the colored curves, the dark blue curve (*R*_0_ = 0.5 μm) exhibits the highest magnitude. Within the studied range, only the dark blue curve remains consistently above the black dotted curve, which represents the Multi-bubble with different radii model. Conversely, the yellow curve (*R*_0_ = 3 μm) has the lowest magnitude, remaining below the black dotted curve throughout. Notably, the dark blue curve (*R*_0_ = 0.5 μm) shows minimum variation with increasing bubble quantity, maintaining a nearly constant value around 237 kPa. The red curve (*R*_0_ = 1 μm) remains stable at around 170 kPa with increasing bubbles and little variation. This is because, for bubbles with *R*_0_ = 0.5 μm and *R*_0_ = 1 μm, the bubble volume is small. In the multi-bubble with the same radius model, when *R*_0_ = 1 μm, even with the maximum *Number* of 6 × 10^5^ bubbles, the ratio of total bubble volume to the computational region volume is only 0.0093 %. The sparse bubble distribution minimizes bubble–bubble interactions, allowing the system to be approximated as a single-bubble vibration case, resulting in a nearly constant cavitation threshold. However, the yellow curve (*R*_0_ = 3 μm), with a slightly larger bubble radius, exhibits more noticeable variation. When the total number of bubbles is less than 2.1 × 10^5^, the yellow curve fluctuates and rises with the increase of bubble count. When *Number* = 2.1 × 10^5^, the cavitation threshold reaches more than 120 kPa. Then it shows a slightly downward trend, with the increase of *Number*. When the bubble count exceeds 5 × 10^5^, it has no significant impact on the cavitation threshold. For purple (*R*_0_ = 6 μm), green (*R*_0_ = 8 μm) and light blue (*R*_0_ = 10 μm), curves fluctuate more prominently. Bubbles with these radii are larger in volume and closer in the bubble cluster. Thus, changes in the number of bubbles affect them more considerably. Then we analyze the black dotted curve representing the Multi-bubble with different radii model: when the total number of bubbles is less than 3 × 10^5^, the black dotted curve shows an upward trend with the increase of *Number*, that is, the larger the *Number*, the larger the cavitation threshold calculated by the Multi-bubble with different radii model. When *Number* = 3 × 10^5^, the cavitation threshold can reach more than 220 kPa. When *Number* continues to increase, the cavitation threshold shows a slight decline before stabilizing. When *Number* is greater than 5 × 10^5^, it has no significant effect on the cavitation threshold. Above all, these results indicate that a larger bubble population does not necessarily correspond to a higher cavitation threshold. A possible reason is that, as mentioned in our previous work [46], the acoustic pressure radiated by the bubble cluster does not vary monotonically with the increase in the number of bubbles, which consequently leads to a non-monotonic relationship between cavitation threshold and bubble population.

### 3.6 Summary and discussion

From the analysis of the effect of each parameter on the cavitation threshold in Section 3, it can be observed that in comparison, the cavitation threshold is the most sensitive to changes in frequency. Observing Fig. 2, when the frequency increases from 500 kHz to 2500 kHz, the corresponding cavitation threshold will increase by approximately 1000 kPa (*R*_max_ ≥ 2*R*_0_ criterion), while in actual therapeutic ultrasound, the sound wave frequency will reach a higher level. For example, it can reach 3 MHz in Histotripsy and 5 MHz in HIFU [55]. Correspondingly, the cavitation threshold will also increase to a higher value. If calculated based on the parameters in Table 2, when the frequency is 5 MHz, the cavitation threshold in the viscoelastic medium will reach 2843 kPa. At this point, the variation range is approximately 2300 kPa. The influence of viscoelasticity is demonstrated in Fig. 13(a). By selecting the cavitation thresholds corresponding to the viscosity range of 0–20 mPa·s and the shear modulus range of 0–200 kPa (which can cover most biological tissues [56]) in Fig. 13(a) for analysis, it can be obtained that the range they span is approximately 1300 kPa. Observe the black dotted line in Fig. 14, within the range of the number of bubbles in this study, the value of the cavitation threshold is approximately 120 kPa-240 kPa, with a variation range of only 120 kPa.

Whether applied to ultrasonic therapy or industrial processing, the selection and optimization of ultrasonic parameters of cavitation signal are of vital importance.

When using the ultrasonic cavitation effect for treatment, optimizing the ultrasonic parameters can improve the therapeutic effect and ensure safety. In this case, multiple factors such as the acoustic characteristics of the signal and the tissue characteristics of the patient need to be considered comprehensively. It is first necessary to assess the characteristics of the tissue to obtain information such as tissue hardness, water content, vascular distribution and position. Adjust the ultrasonic parameters based on the sound velocity, attenuation coefficient and impedance data of the tissue. The frequency adopted is determined by the depth of the target. Low-frequency signal penetrates deeper and has a lower cavitation threshold. When applying to the treatment of deep tissues, the dose needs to be carefully controlled to avoid damage to non-target areas. High-frequency signal has shallow penetration and is suitable for the treatment of superficial tissues. However, its cavitation threshold is higher and a greater excitation sound pressure is required. The required frequency needs to be comprehensively considered based on the specific conditions of different patients. Of course, it is also related to the characteristics of the tissues along the sound wave propagation path. If there are tissues that have a strong attenuation effect on sound waves, a lower frequency is needed to adjust the penetration depth. For the determination of the amplitude of the signal, we should first ensure the approximate range of the cavitation threshold by pre-experiments or numerical calculations. The sound pressure is adjusted to be slightly below the cavitation threshold. While monitoring in real time, gradually increase the amplitude of the sound pressure to excite the cavitation effect. The sound pressure should be precisely controlled to avoid unnecessary damage caused by overly intense inertial cavitation. Short pulses (such as 1–5 cycles) and long intervals (duty cycle 5 %-20 %) are adopted to reduce heat accumulation and damage to non-target areas. The mechanical effect of cavitation is utilized to destroy the target. The pulse repetition frequency (PRF) is adjusted according to the duration of cavitation bubbles to ensure that most bubbles can dissipate during the intervals.

When using the ultrasonic cavitation effect for industrial processing, the matching of material properties and ultrasonic parameters is the key to improving the processing efficiency and quality. The ultrasonic frequency of cavitation signal used in industrial processing is usually lower than that in treatment. The ultrasonic parameters should be reasonably selected by comprehensively considering the characteristics such as material density, hardness and heat sensitivity. Dealing with high-hardness materials usually requires high-power, low-frequency signals to enhance the mechanical effect of cavitation. For low-density or brittle materials, the intensity of cavitation needs to be controlled to avoid excessive damage. For heat-sensitive materials, the processing time should be shortened or a pulse mode should be adopted to avoid material denaturation caused by the high-temperature effect of cavitation. The power or duration of the signal should be adjusted according to the material's tolerance to avoid damaging the material.

## 4 Conclusion and outlook

In this paper, the cavitation threshold in both water and viscoelastic medium is calculated using different criteria based on bubble radius, bubble wall velocity and internal bubble temperature. The results are compared across different models, including the models of Multi-bubble with the same radius and Single bubble, to assess the influence of different bubble models, bubble number and viscoelasticity of the medium. The results are also compared with the Blake cavitation threshold and previous experimental findings.

From the calculation, we find that there is a *P* = A*f* ^α^ *+* B relationship between cavitation threshold and ultrasonic frequency. A, B and α are related to the medium properties and different cavitation judgment criteria. The Blake cavitation threshold is closer to the numerical calculation results at low frequencies (about 20 kHz). For smaller bubble sizes (*R*_0_ = 0.5 μm and *R*_0_ = 1 μm), the cavitation thresholds calculated using the Multi-bubble with the same radius and Single bubble models exhibit minimal change with frequency, and increase as the bubble radius enlarges. Due to the combined effects of the medium and the complexity of the bubble response, the cavitation threshold in viscoelastic media is not always higher than that in water, and it does not follow a simple trend with increasing shear modulus. Furthermore, as the number of bubbles increases, the cavitation threshold exhibits a non-monotonic trend, initially increasing before stabilizing. The cavitation threshold is more sensitive to the change of frequency. The frequency is an important parameter in treatment. Besides being related to the cavitation threshold, it is also closely related to the depth and size of the target object. It is necessary to conduct in-depth research on a more precise correspondence between frequency and cavitation threshold in the future.

Although the models used in this study do not perfectly replicate real-world conditions, the Multi-bubble model with different radii model more closely approximates the cavitation bubble clusters that occur in reality compared to the Single-bubble and the Multi-bubble with the same radius models. The insights derived from the relevant numerical calculations can provide guidance for the subsequent estimation and analysis of the cavitation threshold. Future studies can enhance the theoretical model by considering factors such as the non-spherical deformation of bubbles and the excitation sound pressure in the form of pulse waves, which are closer to the real world conditions. Based on these improvements, experiments can be designed to compare the numerically calculated cavitation thresholds, with the aim of identifying the appropriate range of cavitation thresholds for specific applications.

## CRediT authorship contribution statement

**Xiaozhuo Shen:** Writing – original draft, Software, Investigation, Formal analysis. **Pengfei Wu:** Writing – review & editing, Methodology, Conceptualization. **Weijun Lin:** Writing – review & editing, Supervision.

## Declaration of competing interest

The authors declare that they have no known competing financial interests or personal relationships that could have appeared to influence the work reported in this paper.
